# Comparison Between Water Aerobics and Deep-Water Running on Middle-Aged Adults’ Anthropometric, Hemodynamic and Functional Outcomes

**DOI:** 10.3390/ijerph22010106

**Published:** 2025-01-14

**Authors:** Larissa dos Santos Leonel, Angelica Danielevicz, Rodrigo Sudatti Delevatti

**Affiliations:** Programa de Pós-Graduação em Educação Física, Centro de Desportos, Universidade Federal de Santa Catarina, Campus Universitário, Florianópolis 88040-900, SC, Brazil; larissa.leonel@posgrad.ufsc.br (L.d.S.L.); angelica.danielevicz@posgrad.ufsc.br (A.D.)

**Keywords:** aquatic exercise, physical fitness, exercise, aerobic training, water-based exercise

## Abstract

Background: Head-out aquatic training, using modalities such as water-aerobics/hydrogymnastics (HYD) and deep-water running (DWR), has been effective in improving the physical, metabolic and cognitive health of middle-aged adults. However, direct comparisons between these modalities are lacking. Aim: The aim of this study was to compare the effects of water aerobics and deep-water running on anthropometric, functional and hemodynamic outcomes in adults and older adults. Methods: An uncontrolled pragmatic trial (RBR-2txw8zy) was conducted with participants aged 30 to 80, allocated to HYD and DWR groups. The intervention consisted of 12 weeks of progressive aerobic training with weekly undulating periodization (2× week), divided into three mesocycles (4, 5, and 3 weeks), each lasting 50 min. Intensity was prescribed using the Rate of Perceived Effort (RPE), ranging from RPE 11 to 17. Outcomes assessed included the 30 s chair stand, 30 s arm curl, Timed-Up-and-Go usual (TUG-u) and maximum (TUG-m), 6 min walking test (6MWT), body mass, waist circumference, blood pressure and resting heart rate-HRrest. The analysis was conducted using generalized estimating equations, with per-protocol (PP) and intention-to-treat (ITT) analyses. Results: The study included 104 participants (HYD: *n* = 63, mean age 59 years, 54 women; DWR: *n* = 41, mean age 53 years, 33 women). ITT analysis showed improvements in waist circumference, waist-to-height ratio, and TUG-m in the HYD group, and a reduction in HRrest in the DWR group. Both modalities showed significant improvements in the 30 s chair stand, 30 s arm curl, 6MWT, waist circumference, and waist-to-height ratio in the PP analysis. Conclusions: Both modalities promoted functional improvements and favorable changes in anthropometric evaluations, with DWR showing a greater reduction in HRrest.

## 1. Introduction

Aerobic training is widely recommended due to its demonstrated benefits on the health outcomes of adults and older adults [1] and as a predictor of reduced premature all-cause mortality [2]. Aquatic-based training in an upright position has emerged as an alternative form of aerobic exercise prescription, showing beneficial results for the health of adults and older adults [3,4,5,6,7,8,9]. Additionally, it has shown good adherence rates and, due to the reduced impact on lower limb joints, allows for increased intensity and/or volume of training with less joint overload, consequently reducing the risk of injuries [10].

Among the forms of aquatic exercise in the upright position, hydrogymnastics (HYD) and deep water running (DWR) stand out, practiced in shallow and deep water, respectively. Although both are performed in the upright position, they have some distinctive characteristics. Hydrogymnastics is a modality conducted in immersion at a depth reaching the xiphoid process, with the feet touching the pool floor, with a reduction of approximately 60–70% in hydrostatic weight [10] and minimal vertical ground reaction force [11,12]. This practice involves a combination of lower limb movements in a closed kinetic chain, accompanied by upper limb movements. Due to the nature of the modality and its movements, the displacement is predominantly vertical, and evidence points to lower muscle activation and force production compared to dry land exercises performed at the same cadence [13,14,15].

In contrast, DWR involves walking and/or running submerged in water at shoulder level, using a flotation device to prevent the feet from touching the pool floor, thereby eliminating vertical ground reaction force [16,17]. It simulates running movement patterns found on dry land, but in an open kinetic chain, with the technique involving a slight forward lean of the torso, and circular, cyclical arm and leg movements to achieve horizontal displacement in the water [18]. Due to the nature of the modality and its movement technique, the center of buoyancy is altered [17], which results in greater activation and coordination of the trunk muscles [19].

Given the complexity of the technique, the depth of immersion, and the emphasis on horizontal displacement with increased drag force, this exercise requires substantial engagement of large muscle groups in the lower limbs [20], and it also leads to higher energy expenditure compared to dry land exercises [21]. Overall, systematic reviews and clinical trials report improvements in cardiorespiratory fitness and lower limb muscle strength [3,22], functional capacity [4,7,23], as well as hemodynamic [24] and metabolic benefits [23,25] with regular hydrogymnastics practice. Similarly, improvements in cardiorespiratory fitness [5,6,8,26], functional capacity [6,8], lower limb strength, hemodynamic reductions [6], and metabolic and cognitive benefits in women [27] have been evidenced with DWR practice.

Despite their similar health benefits for adults and older adults, these modalities exhibit certain differences, such as the prioritized kinetic chains, the predominant type of displacement in the aquatic environment (vertical or horizontal), and the varying levels of stabilization and complexity in their techniques. These factors influence movement performance, muscle recruitment, and possibly energy expenditure, which may result in differing magnitudes of effects on participants of each modality. In this context, no studies to date have compared the impact of hydrogymnastics and deep-water running on anthropometric, functional, and hemodynamic outcomes in adults and older adults practicing these activities. Therefore, the present study aims to compare the effects of these two modalities on anthropometric measurements, functional fitness, and hemodynamic aspects in adults and older adults. We hypothesize that both modalities will have similar changes in body composition, hemodynamic outcomes, functional mobility and upper limb strength with the possibility of superiority in cardiorespiratory and lower limb strength for DWR.

## 2. Methods

### 2.1. Trial Design

This is an uncontrolled, non-randomized pragmatic trial, that analyzed two intervention groups with aquatic training in the upright position, conducted in parallel (hydrogymnastic and deep-water running). A convenience sampling method was used, in which participants selected modalities (HYD or DWR) they were going to practice. The protocol was registered in the Brazilian Registry of Clinical Trials (RBR-2txw8zy).

### 2.2. Participants

Participants of the Vertical Aquatic Activities Extension Program—Floripa Aquatic Training and Health Outcomes (FATHO), affiliated with the Federal University of Santa Catarina (UFSC), enrolled in the first semester of 2024 were invited to participate in this study. The following inclusion criteria were considered: being between 30 and 80 years old, of both sexes, and having medical authorization for physical exercise. The exclusion criterion was the presence of functional limitations that prevented participants from performing the tests at baseline. All participants signed the Informed Consent Form, and the study was approved by the Human Research Ethics Committee (5.510.243).

### 2.3. Interventions

The training programs were conducted at the aquatic complex of the Sports Center at UFSC, with sessions led by trained professionals and students from the Physical Education course at the institution. The hydrogymnastics group (HYD) used an adapted pool with a depth of immersion up to the xiphoid process, with a temperature ranging from 28 to 31 °C. Meanwhile, the deep-water running group (DWR) trained in an Olympic pool, with immersion depth at shoulder and neck level, using flotation vests at waist height, with a temperature ranging from 26 to 28 °C.

Both groups performed the same periodization, differing only in modality (HYD and DWR). The protocol consisted of a 12-week macrocycle of progressive aerobic training with weekly undulating periodization, including two sessions per week (on Mondays and Wednesdays) with a total duration of 44 to 50 min. Each session comprised 5 min of warm-up, 36 to 42 min of main activity, and 3 min of cool-down. The macrocycle was structured into three mesocycles: the first mesocycle lasted four weeks, the second lasted five weeks, and the last three weeks. In the transition from the first to second mesocycle, volume was increased (one block of 6 min), and in the transition from the second to third mesocycle, the intensity was increased. The intensity of aerobic training was prescribed using the Borg Rating of Perceived Exertion (RPE) scale, ranging from 6 to 20 [28], progressing from RPE 11 (light) to 17 (very hard). On Mondays, continuous methods were used, while on Wednesdays, pyramid methods were employed.

The HYD group performed the following exercises: (1) stationary running, (2) posterior elevation, (3) lateral glides, (4) frontal glides, (5) frontal kicks, and (6) posterior running. In the second mesocycle, a pool noodle was used in Monday classes, and in the third mesocycle, the equipment was switched to aqua dumbbells. In the DWR group, the movement techniques for the upper limbs were alternated on Mondays, with the arms submerged in front, involving pit movements from bottom to top. More information about the periodization model for both groups can be found in Table 1.

### 2.4. Outcomes

To characterize the participants, an initial anamnesis was conducted to collect sociodemographic information (age and sex), health conditions (health history and medication use), and experience with the modalities and other physical exercises.

The primary and secondary outcomes were assessed before and after the 12-week intervention. As the primary outcome of the study, functional fitness was assessed through the Rikli and Jones test battery [29]. For the evaluations, the participants were instructed to wear suitable clothes and received instructions on how to perform each test. Once familiarized, the evaluation was performed following the sequence mentioned above. In summary, resistance and strength of the dominant upper limb were assessed through a 30 s elbow flexion (4 kg for males and 2 kg for females), resistance and strength of the lower limbs were measured through the 30 s chair-stand test, functional mobility was assessed through the Time Up and Go (TUG) test in two speed (maximum speed and habitual), and cardiorespiratory endurance was evaluated using the 6 min walk test (6MWT).

The secondary outcomes are anthropometric and hemodynamic measurements. Body mass and height were measured using a digital scale with a precision of 100 g (Marte^®^, model PP 180, Santa Rita do Sapucaí, Brazil) and a stadiometer with a precision of 1 mm (AlturaExata^®^, Belo Horizonte, Brazil), respectively. In addition, the waist-to-height ratio was also calculated using the ratio between waist circumference (cm) and height (cm). Hemodynamic measurements, including resting systolic (SBP) and diastolic (DBP) blood pressure and resting heart rate, were taken using automatic equipment (OMRON, model HEM-7113, São Paulo, Brazil) after the participants remained at rest, sitting in a calm environment, for 10 min.

### 2.5. Statistical Analysis

To verify differences in the baseline characteristics of participants and adherence, the chi-square test was used for categorical variables. For continuous variables, the Shapiro–Wilk normality test was applied to assess the normality of the model residuals, followed by the Student’s *t*-test for normally distributed data. Continuous variables were expressed as mean and standard deviation and categorical variables as absolute and relative frequency.

To verify the effect of the intervention, the analysis was conducted using both per protocol (PP) and intention-to-treat (ITT) methods. For the PP analysis, only data from participants with both pre- and post-intervention values for the variable of interest and adherence ≥ 70% in the sessions were considered. In contrast, the ITT analysis included all participants, and for those with missing data in post-intervention assessments, maximum likelihood was performed. Generalized Estimating Equations (GEEs) were employed for these analyses (effect time, group and time*group), utilizing the Bonferroni post-hoc test. The significative effect group (g) indicates the difference between the hidrogymnastic (HYD) and deep-water running (DWR) groups regardless of time; the significative effect time (t) indicates the difference between pre- and post-intervention values; and the group*time interaction (g*t), if significant, suggests that the time effect (pre- vs. post-intervention) differs between the groups. The data are expressed as means and standard errors. The significance level adopted was *p* ≤ 0.050. Furthermore, the difference from post to pre-intervention and the 95% confidence interval will be presented to understand the magnitude of the intervention effect. All analyses were performed using SPSS version 23.0 (IBM Corp., Armonk, NY, USA). Additionally, no sample size calculation was performed.

## 3. Results

**Figure 1:**
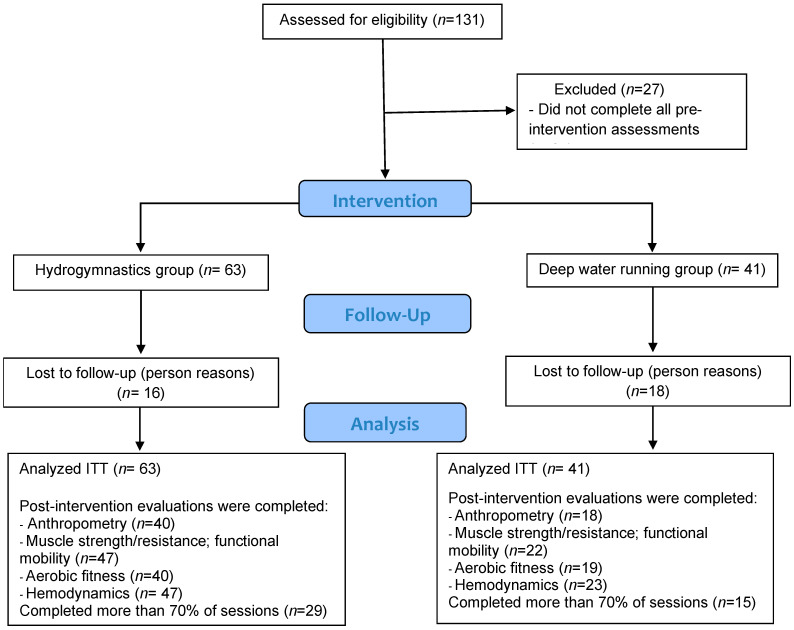
Flow diagram.

A total of 131 participants volunteered for the study, with 27 participants excluded for not completing all pre-intervention assessments. Consequently, 104 participants were included in the study, with 63 participants in the HYD group and 41 in the DWR group. Of these, 42.2% (*n* = 27) and 31.7% (*n* = 13) were students from the previous semester in their respective modalities (*p* = 0.253). During the follow-up, there was a sample loss of 25.4% (*n* = 16) in the HYD group and 43.9% (*n* = 18) in the DWR group (*p* = 0.080) (Figure 1).

Among the 104 participants evaluated, the majority were women and 45 were older adults. There were no significant differences between the groups in terms of sociodemographic data, presence of chronic diseases, or medication use. The most used medications were antidepressants, statins, and thyroid preparations. Additionally, approximately half of the participants reported engaging in other forms of physical activity besides upright position aquatic training (Table 2). The most frequently reported activities were walking (21 participants) and Pilates (12 participants).

The mean adherence of the HYD participants was 68.68% ± 20.39% and the DWR had an adherence of 58.23% ± 27.39% (*p* = 0.033). The aquatic training was well tolerated by the participants, with no adverse events occurring.

**Figure 2:**
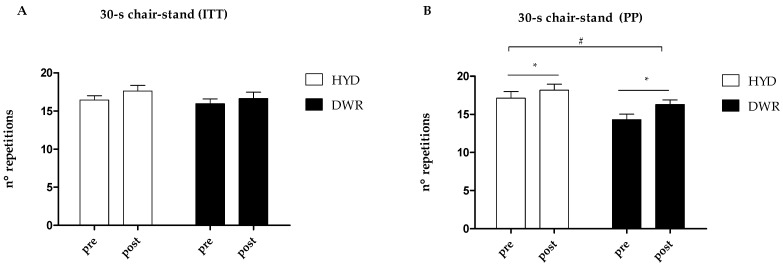
Effects of 12 weeks of hydrogymnastic and deep-water running in 30 s chair-stand test. (**A**) 30-s Chair Stand (ITI); (**B**) 30-s Chair-Stand (PP). Note: DWR: deep water running group; HYD: hydrogymnastics group; ITT: intention-to-treat analysis; n^o^: number; PP: per-protocol analysis; data are presented as mean ± standard error; intra and intergroup comparisons were performed using generalized estimation equations with Bon-ferroni post hoc; *: significant intra-group differences (*p* ≤ 0.050). #: indicate a difference between the groups (*p* ≤ 0.050).

In Figure 2, regarding the functional outcome of the 30 s chair-stand test, it can be observed that there were no significant changes in the groups after 12 weeks of intervention (*p* value: g = 0.405; t = 0.060; g*t = 0.633) in the ITT analysis (Figure 2A), with the magnitude of change in HYD (95% CI: 1.17, −0.22; 2.57) and in DWR (95% CI: 0.68, −0.73; 2.11). However, in the PP analysis (Figure 2B), a significant improvement was shown in both groups over time (*p =* 0.001). Additionally, the groups were different at baseline and at 12 weeks (*p =* 0.014).

**Figure 3:**
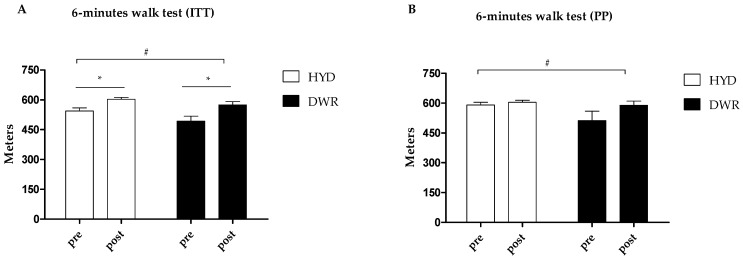
Effects of 12 weeks of hydrogymnastic and deep-water running in 6 min walk test. (**A**) 6-min walk test (ITT); (**B**) 6-min walk test (PP). Note: DWR: deep water running group; HYD: hydrogymnastics group; ITT: intention-to-treat analysis; PP: per-protocol analysis; data are presented as mean ± standard error; intra and intergroup comparisons were perfermed using generalized estimation equations with Bon-ferroni post hoc; *: significant intra-group differences (*p* ≤ 0.050). #: indicate a difference between the groups (*p* ≤ 0.050).

In Figure 3, regarding the 6 min walk test, it can be observed in the ITT analysis (Figure 3A) that both groups increased the distance covered (HYD: 95% CI = 59.77, 29.09; 90.44 and DWR: 95% CI = 81.40, 31.63; 131.17; *p* value t ≤ 0.001). However, the groups were different at baseline and at 12 weeks (*p* = 0.040). When analyzing only those who completed ≥70% of the sessions, it was observed that both groups improved (HYD: 95% CI = 13.62, −13.25; 40.49 and DWR: 95% CI = 77.24, 4.21; 150.27; *p* value t = 0.022).

Table 3 describes anthropometric measurements, functional fitness and hemodynamic results. In anthropometric measurements, significant improvements were observed in the waist circumference and waist-to-height ratio in HYD in ITT analysis and in both groups in PP analysis. Regarding functional fitness, the TUG-m showed a significant improvement in both groups in the PP analysis, while TUG-m showed significant improvements only in HYD in ITT analysis. The 30 s arm curl shows improvements in both groups and in both analyses. Hemodynamic outcomes showed a significant time effect, with both groups showing an increase in systolic blood pressure in the ITT analysis and a significant improvement in DWR in both analyses in resting heart rate.

## 4. Discussion

The aim of the present study was to compare the effects of 12 weeks of hydrogymnastics and deep-water running on anthropometric measurements, functional fitness, and hemodynamic outcomes in middle-aged adults. The main findings included improvements in waist circumference, waist-to-height ratio, and the TUG-m in the HYD group, as well as a reduction in resting heart rate in the DWR group. Additionally, both modalities showed significant improvements in the 30 s arm curl test and the 6 min walk test.

The hydrogymnastics group showed greater adherence to the training program compared to the DWR, which may have influenced the results of the ITT analysis. We speculate that this difference in adherence could be partly due to several factors, such as the water temperature being 3 to 5 °C lower in DWR, and even the participants’ unfamiliarity with the modality. Another point is that the HYD included equipment to help alleviate monotony, as noted in previous reports. The training was safe for participants, with no adverse effects observed. Additionally, participants who attended more than 70% of the sessions showed improvements in the 30 s chair stand test, 30 s arm curl test, TUG-m, and in the 6MWT in both groups. It is important to note that, even for measures that did not show statistically significant improvements after 12 weeks, there was a maintenance of pre-intervention values, with no worsening in any anthropometric, functional, or hemodynamic markers. Given the heterogeneity of the participants, who are younger, trained, and without associated comorbidities, this maintenance is even more interesting.

In terms of anthropometric measurements, in ITT analysis, the HYD group showed a decrease of 6.11 cm in waist circumference and a reduction of 0.04 in the waist-to-height ratio, while practicing at least 70% of the sessions resulted in reductions over time in both groups, this indicates that both modalities, when practiced with higher adherence, benefit body composition. Evidence suggests that reductions in waist circumference are more pronounced when training is conducted at higher volumes and adherence levels [30,31]. Furthermore, these reductions are important, as waist circumference and waist-to-height ratio are associated with all-cause and cardiovascular mortality [27,28]. A systematic review by Carmienke et al. [32] demonstrated that waist circumference was associated with increased all-cause mortality above values of 95 cm for men and 80 cm for women. Furthermore, a 2022 cohort study [33] showed that the waist-to-height ratio was associated with cardiovascular disease risk in people with hypertension and had better predictive value for cardiovascular incidence compared to waist circumference or BMI.

In the functional fitness outcomes, the HYD group showed an improvement in the TUG-m, and both groups showed improvements in the 30 s arm curl test and the 6 min walk test in the ITT analysis, as well as in the 30 s chair stand test in the PP. These results align with other studies that have found functional benefits for adults and older adults who performed aquatic training, such as improvements in cardiorespiratory adaptations, dynamic balance and muscle strength [7,22,34,35]. The aging process leads to declines in muscle function and cardiorespiratory fitness, which impair the ability to perform daily activities and live independently [1]. The results of the present study reinforce the importance of exercise for maintaining quality of life in old age [36].

Regarding the hemodynamic outcomes, in the ITT analysis, the results indicate significantly higher systolic blood pressure values after 12 weeks; however, when observing those who performed at least 70% of the sessions, there were no changes in SBP. A meta-analysis by Igarashi and Nogami [37] identified that upright position aquatic exercises and swimming significantly reduce systolic and diastolic blood pressure, differing from the findings of the present study. However, it is important to note that the mean values for both groups at baseline and after 12 weeks can be considered optimal (<120 mmHg) according to the Brazilian Guidelines for Blood Pressure Measurement Inside and Outside the Ambulatory Settings [38], in addition to the fact that the sample predominantly consisted of normotensive individuals. To reinforce this, a systematic review and meta-analysis were performed by Reichert et al. [39], in which aquatic training in an upright position demonstrated that reductions in blood pressure are significant only in hypertensive individuals.

Furthermore, the higher values observed after the intervention may have been influenced by the data collection period, as the initial samples were taken during the summer and the subsequent ones during the winter. Studies suggest that seasonal variation affects blood pressure response, with a tendency for higher values in winter [40,41]. A population-based study involving 16 countries, with men and women aged 35–64 years, observed an average increase of up to 2.06 mmHg in SBP during midwinter compared to midsummer [42]. However, it is important to note that these changes in SBP are more pronounced at lower temperatures.

On the other hand, the decrease in resting heart rate in the DWR group in both analyses represents an important indication of improvement in cardiorespiratory health. Resting heart rate is a crucial indicator of cardiac output and predicts longevity and cardiovascular diseases, serving as an important marker for outcomes in conditions like heart failure [43]. In addition, this superior effect on DWR may be associated with the depth of immersion at which the activity was performed, as greater depths are linked to increased bradycardia [44].

It is worth noting that, despite the temperature variation in the two modalities (HYD: 28–31 °C; DWR: 26–28 °C), it is unlikely that it influenced the hemodynamic differences, as both modalities showed similar values, especially in blood pressure, and the pool temperatures were within the thermoneutral range (28–32 °C) [45]. Evidence indicates that extreme temperatures (below 20 °C or above 33 °C) induce different hemodynamic adaptations, influenced by immersion time and water depth [46,47,48,49]. For example, Šrámek et al. [49] reported that immersion at 14 °C for 60 min increased heart rate by 3 bpm and blood pressure by 8 mmHg (SBP) and 6 mmHg (DBP). In contrast, temperatures of 20 °C and 32 °C showed similar reductions in heart rate and blood pressure. Pallubinsky et al. [47] found that temperatures above 36 °C for over 60 min increase heart rate.

The present study has some limitations inherent to the pragmatic nature of the trial, such as the lack of a control group, the absence of participant randomization, and also the failure to carry out a prior sample calculation. Additionally, since this is a community-based project, the participants present a wide heterogeneity, which may lead to challenges in sample characterization. However, the project aims to improve the overall health of individuals representing a cross-section of adults and older adults who are currently active in society, while offering the opportunity for the entire community to engage in physical activity in an equitable manner. In terms of strengths, it is noteworthy that this is the first study to compare the two most widely practiced upright position aquatic modalities globally in terms of functional, hemodynamic, and anthropometric outcomes.

## 5. Conclusions

It is concluded that both the HYD and DWR modalities result in functional improvements in participants, with particular emphasis on the 30 s arm curl test and the 6 min walk test. Additionally, the HYD modality appears to be superior in anthropometric assessments, while DWR shows greater improvement in resting heart rate. However, despite the statistical differences, the values are not markedly divergent between the groups, favoring the overall health of participants in a similar manner. Notably, the HYD modality showed better adherence to the training program, which may lead to more pronounced results over the same period of time.

## Figures and Tables

**Table 1:** Periodization model for both groups.

Mesocycle	Weeks	Mondays (Continuous)	Wednesday (Pyramid)	Duration Main Part	Total Duration
I	1–4	RPE 11	6× RPE 11-13-15 (3:2:1)	36 min	44 min
II	5–9	RPE 11	7× RPE 11-13-15 (3:2:1)	42 min	50 min
III	10–12	RPE 13	7× RPE 13-15-17 (3:2:1)	42 min	50 min

Note: RPE: Rating of perceived exertion. The information between parentheses is time in minutes in each intensity.

**Table 2:** Characterization of the participants (*n* = 104).

Variables	HYD (*n* = 63)	DWR (*n* = 41)	*p* Value
Age (years)	59 (47–65)	53 (41–64)	0.329
Sex (female)	54 (85.7%)	33 (80.5%)	0.665
Diabetes mellitus	3 (4.7%)	3 (7.3%)	0.676
Dyslipidemia	16 (25.3%)	13 (31.7%)	0.869
Systemic arterial hypertension	18 (28.5%)	10 (24.4%)	0.657
Practice of other physical exercises	31 (49.2%)	22 (53.6%)	0.902
ACE inhibitors	4 (6.3%)	4 (9.8%)	0.524
ANG-II antagonist	9 (14.3%)	1 (2.4%)	0.045
Antidepressant	8 (12.7%)	5 (12.5%)	0.940
Antiepileptics	3 (4.8%)	2 (4.9%)	0.978
Antipsychotics	3 (4.8%)	1 (2.4%)	0.547
Antirheumatic	3 (4.8%)	0 (0.0%)	0.156
Beta-blocker	4 (6.3%)	1 (2.4%)	0.362
Biguanide antidiabetics	1 (1.6%)	1 (2.4%)	0.757
Diuretics	3 (4.8%)	3 (7.3%)	0.585
Hmg-Coa reductase inhibitors	4 (6.3%)	3 (7.3%)	0.847
Non-Barbiturates Sedative/Hypnotics	1 (1.6%)	1 (2.4%)	0.757
Proton pump inhibitors	2 (3.2%)	2 (4.9%)	0.659
Statins	7 (11.1%)	2 (4.9%)	0.269
Thyroid preparations	15 (23.8%)	6 (14.6%)	0.255

Note: DWR: deep water running group; HYD: hydrogymnastics group. Continuous non-parametric data are presented as the median and interquartile range (P25–P75). Categorical data are presented as absolute frequency (sample n) and relative frequency (%).

**Table 3:** Effects of 12 weeks of hydrogymnastic and deep-water running on health outcomes in middle-aged adults.

Outcomes (Analysis)	Group (*n*)	Baseline $\underline{x}$ ± se	12 Weeks $\underline{x}$ ± se	Δ (95% CI)	Value *p*		
					g	t	g*t
Anthropometric measurements							
Body mass (ITT)	HYD	72.39 ± 1.98	70.30 ± 2.33	−2.00 (−5.21; 1.02)	0.611	0.710	0.134
	DWR	73.40 ± 2.31	74.80 ± 3.51	3.36 (−3.11; 10.07)			
Body mass (PP)	HYD (*n* = 46)	72.65 ± 2.84	73.27 ± 2.90	0.62 (−0.47; 1.72)	0.990	0.879	0.351
	DWR (*n* = 24)	73.11 ± 5.57	72.66 ± 4.83	−0.44 (−2.41; 1.51)			
Body mass index (ITT)	HYD	27.57 ± 0.65	28.43 ± 1.36	0.85 (−1.30; 3.00)	0.825	0.236	0.925
	DWR	27.35 ± 0.76	28.08 ± 1.15	0.72 (−0.75; 2.21)			
Body mass index (PP)	HYD (*n* = 46)	27.70 ± 1.12	27.89 ± 1.10	0.19 (−0.19; 0.58)	0.882	0.832	0.426
	DWR (*n* = 24)	27.59 ± 1.46	27.48 ± 1.24	−0.11 (−0.76; 0.53)			
Waist circumference (ITT)	HYD	94.10 ± 1.80	87.98 ± 2.19 *	−6.11 (−9.21; −3.00)	0.802	0.042	0.020
	DWR	91.56 ± 1.96	91.98 ± 2.98	0.41 (−4.12; 4.95)			
Waist circumference (PP)	HYD (*n* = 46)	94.78 ± 2.79	91.43 ± 2.88 *	−3.35 (−5.86; −0.83)	0.853	0.001	0.393
	DWR (*n* = 24)	93.22 ± 3.73	91.27 ± 3.78 *	−1.95 (−3.93; 0.03)			
Waist-to-Height Ratio (ITT)	HYD	0.58 ± 0.01	0.54 ± 0.01 *	−0.04 (−0.05; −0.01)	0.923	0.084	0.028
	DWR	0.56 ± 0.01	0.56 ± 0.01	0.00 (−0.02; 0.03)			
Waist-to-Height Ratio (PP)	HYD (*n* = 46)	0.58 ± 0.01	0.56 ± 0.01 *	−0.02 (−0.03; −0.00)	0.857	0.001	0.335
	DWR (*n* = 24)	0.57 ± 0.02	0.56 ± 0.02 *	−0.01 (−0.02; 0.00)			
Functional Fitness							
30-s arm curl (ITT)	HYD	19.52 ± 0.57	23.88 ± 0.67 *	4.35 (3.14; 5.57)	0.820	<0.001	0.562
	DWR	19.70 ± 0.55	23.29 ± 1.19 *	3.58 (1.27; 5.89)			
30-s arm curl (PP)	HYD (*n* = 29)	19.44 ± 0.73	24.24 ± 0.68 *	4.79 (3.53; 6.05)	0.663	<0.001	0.455
	DWR (*n* = 13)	19.53 ± 1.23	22.92 ± 1.79 *	3.38 (−0.08; 6.85)			
TUG-h (ITT)	HYD	8.17 ± 1.99	7.70 ± 0.17	−0.46 (−0.80; −0.12)	0.888	0.496	0.257
	DWR	7.92 ± 0.16	8.03 ± 0.48	0.11 (−0.83; 1.06)			
TUG-h (PP)	HYD (*n* = 29)	7.89 ± 0.27	7.74 ± 0.18	−0.15 (−0.83; 0.53)	0.498	0.762	0.744
	DWR (*n* = 13)	7.62 ± 0.20	7.62 ± 0.35	0.00 (−0.63; 0.64)			
TUG-m (ITT)	HYD	6.74 ± 0.17	6.15 ± 0.17 *	−0.58 (−0.80; −0.36)	0.450	0.131	0.027
	DWR	6.57 ± 0.12	6.68 ± 0.30	0.11 (−0.46; 0.69)			
TUG-m (PP)	HYD (*n* = 29)	6.59 ± 0.23	6.18 ± 0.21 *	−0.40 (−1.05; 0.24)	0.826	0.019	0.762
	DWR (*n* = 13)	6.70 ± 0.19	6.17 ± 0.22 *	−0.52 (−0.95; −0.09)			
Hemodynamic							
Systolic Blood Pressure (ITT)	HYD	109.32 ± 2.46	114.00 ± 2.49 *	4.68 (−0.44; 9.80)	0.567	0.036	0.563
	DWR	108.73 ± 1.97	111.39 ± 2.39 *	2.65 (−1.91; 7.22)			
Systolic Blood Pressure (PP)	HYD (*n* = 29)	112.79 ± 2.67	114.29 ± 3.34	1.49 (−5.01; 2.02)	0.646	0.507	0.719
	DWR (*n* = 13)	111.66 ± 2.32	112.11 ± 2.57	0.44 (−4.07; 4.95)			
Diastolic Blood Pressure (ITT)	HYD	72.55 ± 1.18	75.20 ± 1.42	2.65 (0.51; 4.79)	0.270	0.240	0.072
	DWR	72.32 ± 1.26	71.77 ± 1.43	−0.55 (−3.33; 2.21)			
Diastolic Blood Pressure (PP)	HYD (*n* = 29)	74.89 ± 1.83	75.58 ± 2.02	0.68 (−0.98; 2.35)	0.239	0.513	0.177
	DWR (*n* = 15)	73.37 ± 1.96	71.41 ± 1.50	−1.96 (−5.43; 1.49)			
Resting Heart Rate (ITT)	HYD	75.95 ± 1.44	76.24 ± 1.73	0.28 (−2.12; 2.70)	0.968	0.059	0.030
	DWR	78.10 ± 1.50	73.92 ± 1.71 *	−4.18 (−7.42; −0.94)			
Resting Heart Rate (PP)	HYD (*n* = 29)	76.93 ± 1.91	77.94 ± 2.03	1.00 (−1.05; 3.07)	0.305	0.028	0.002
	DWR (*n* = 13)	77.43 ± 3.02	71.25 ± 2.02 *	−6.17 (−10.31; −2.04)			

Note: DWR: deep water running group; HYD: hydrogymnastics group; ITT: intention-to-treat analysis; PP: per-protocol analysis; data are presented as mean ± standard error; intra and intergroup comparisons were performed using generalized estimation equations with Bonferroni post hoc; Δ: difference from post- to pre-intervention; 95% CI: 95% confidence interval; *: significant intra-group differences (*p* ≤ 0.050). Different letters indicate a difference between the groups (*p* ≤ 0.050).

## Data Availability

The original contributions presented in this study are included in the article. Further inquiries can be directed to the corresponding author(s).
